# Association Between the Lactate‐to‐Albumin Ratio and ICU/In‐Hospital Mortality in Critically Ill Patients With Comorbid Type 2 Diabetes Mellitus : A Cohort Study Utilizing the MIMIC‐IV Database

**DOI:** 10.1155/emmi/2751114

**Published:** 2026-04-13

**Authors:** Zhuo-Ting Cheng, Jia-Liang Wang, Yan-Bo Zhao, Zheng-Ming Zhao, Zhi-Jun Li, Yang Liu

**Affiliations:** ^1^ School of Nursing, Hubei University of Medicine, Shiyan, China, hbmu.edu.cn; ^2^ Department of Emergency, Renmin Hospital, Hubei University of Medicine, Shiyan, Hubei, China, hbmu.edu.cn; ^3^ Department of Intensive Care Unit, Yulin Hospital of Traditional Chinese Medicine, Yulin, Shaanxi, China

**Keywords:** lactate-to-albumin ratio, MIMIC database, type 2 diabetes

## Abstract

**Background:**

Type 2 diabetes mellitus (T2DM) accounts for over 90% of diabetes cases worldwide, and its rising prevalence poses a substantial public health challenge. The lactate‐to‐albumin ratio (LAR) is a novel biomarker that reflects both metabolic stress and nutritional‐inflammatory status, and it has demonstrated independent prognostic value in various critical illnesses. However, its association with mortality in critically ill patients with T2DM remains unclear. This study aims to evaluate the predictive value of LAR for prognosis in this specific patient population through a retrospective analysis.

**Methods:**

This retrospective observational cohort study utilized data from the Medical Information Mart for Intensive Care IV (MIMIC‐IV) v3.0 database, which includes complete medical records of patients admitted to the intensive care unit (ICU) of a U.S. medical center between 2008 and 2022. Subjects were categorized into three groups based on LAR tertiles. The study assessed ICU mortality and in‐hospital mortality as primary outcomes; 30‐day, 90‐day, and 365‐day mortality after ICU admission served as secondary outcomes. Machine learning identified key variables related to LAR and outcomes. The association between LAR and primary outcomes was analyzed using multivariate logistic regression and restricted cubic spline (RCS) regression. Cumulative mortality was analyzed using Kaplan–Meier curves. Threshold effects were evaluated using generalized additive models to enhance clinical applicability. Sensitivity and subgroup analyses were used to validate the robustness of the results and the interactions within subgroups.

**Result:**

The study included 5463 critically ill T2DM patients. Using Boruta and SHapley Additive exPlanations (SHAP) algorithms, 14 key variables were identified. RCS analysis revealed a nonlinear relationship between LAR and in‐hospital mortality (*p* for nonlinearity = 0.001). Threshold effect analysis identified a critical LAR threshold of 2.10. Kaplan–Meier survival curves showed that higher LAR values were correlated with increased mortality (*p* < 0.001). Subgroup analyses revealed significant interactions in gender subgroups for ICU mortality (*p* for interaction = 0.043) and in hyperlipidemia (HLD) subgroups for in‐hospital mortality (*p* for interaction = 0.049). Sensitivity analyses confirmed robust associations between LAR and ICU mortality (OR 1.32, 95% CI: 1.20–1.45, *p* < 0.001) and in‐hospital mortality (OR 1.37, 95% CI: 1.26–1.50, *p* < 0.001).

**Conclusion:**

This study identified a significant correlation between elevated LAR and adverse ICU outcomes in critically ill patients with T2DM.

## 1. Introduction

Diabetes is a chronic condition characterized by high blood sugar due to disrupted carbohydrate metabolism [1]. T2DM, the most common form, results from insulin resistance and inadequate insulin production [2], comprising over 90% of diabetes cases globally [3]. Its prevalence is rising due to economic growth and an aging population, affecting 536.6 million adults (10.5% of the global adult population) in 2021 [4, 5]. Identifying reliable biomarkers to predict T2DM progression and guide treatment is crucial for improving outcomes and addressing this growing public health issue.

As the exploration of big data continues to expand, digital biomarkers have become essential components of clinical decision‐making processes. Biomarkers, including the triglyceride‐glucose index, the systolic blood pressure‐to‐heart rate ratio, and measures of glucose variability, have demonstrated significant correlations with the prognostic outcomes of various critical illnesses [6–8]. The LAR emerges as a novel biomarker, derived from the ratio of lactate to serum albumin. Blood lactate is a well‐recognized prognostic indicator in critically ill patients [9, 10]. Previous research has demonstrated a correlation between elevated lactate levels and insulin resistance in adipocytes, as well as the development of T2DM [11]. Furthermore, evidence suggests that serum lactate levels may serve as a predictive marker for the onset of diabetes [12, 13]. Albumin, the most abundant protein in serum, is intricately linked to an individual’s nutritional status and inflammatory response [14]. Research conducted by Succurro et al. has shown that serum albumin levels are frequently significantly reduced in individuals with T2DM, which is considered a risk factor for the onset and progression of the disease [15]. Additional studies by Liu et al. [16] suggest that decreased serum albumin levels can impair insulin sensitivity in hepatocytes among patients with T2DM, thus highlighting the importance of maintaining appropriate albumin levels for improving insulin resistance and overall disease management.

Recent research has identified the LAR as an independent prognostic indicator for elderly patients in critical care and those with severe pneumonia, demonstrating a predictive capacity that exceeds that of either marker when used individually [17, 18]. Numerous retrospective analyses have established that LAR can independently predict short‐term mortality across various critical conditions, such as acute pancreatitis [19], sepsis‐related liver injury [20], acute heart failure (HF) [21], and acute myocardial infarction (MI) [22].

Despite extensive evidence in critical care, there is a notable lack of research on the prognostic value of the LAR in critically ill patients with comorbid T2DM. Patients with T2DM present a unique clinical challenge, characterized by chronic subclinical inflammation, oxidative stress, and metabolic derangements [23], Preexisting conditions can significantly affect tissue perfusion and protein metabolism during acute stress. LAR effectively indicates systemic hypoperfusion (through lactate) and nutritional/inflammatory status (through albumin), making it ideal for assessing the complex health challenges in critically ill T2DM patients.

Our main hypothesis is that a high LAR at admission is linked to higher mortality in critically ill T2DM patients. This study will use a large retrospective public database to assess the prognostic value of LAR in this group, addressing a gap in the literature.

## 2. Materials and Methods

### 2.1. Study Design and Population

This retrospective observational cohort study utilized data from the MIMIC‐IV 3.0 database (https://mimic.mit.edu), a large, single‐center, publicly available clinical database that includes comprehensive medical records of 94,458 patients admitted to the Beth Israel Deaconess Medical Center in Boston, Massachusetts, USA, between 2008 and 2022. Access to the database was granted to one of the authors (Yang Liu) after completing the Collaborative Institutional Training Initiative (CITI) program (Certification Number: 65,970,781). Due to the de‐identified nature of the patient health information in this database, the requirement for ethical approval was waived.

### 2.2. Inclusion and Exclusion Criteria

**FIGURE 1:**
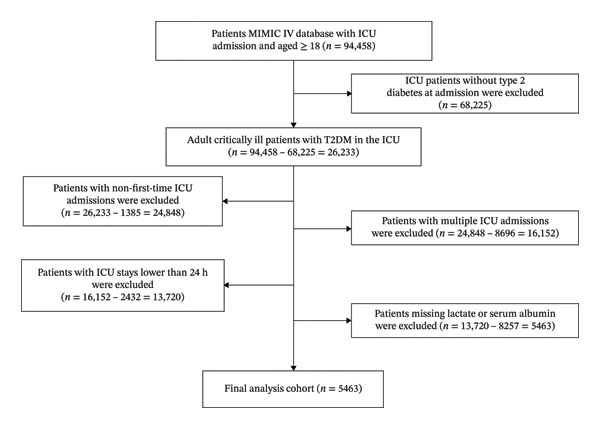
Flowchart of subject selection. MIMIC: medical information mart for intensive care; ICU: intensive care unit.

This study included all critically ill adult patients (aged ≥ 18 years) admitted to the ICU of the Beth Israel Deaconess Medical Center in Boston, Massachusetts, USA, between 2008 and 2022. Patients were excluded if they met any of the following criteria: (1) ICU patients without T2DM at admission were excluded; T2DM diagnosis was based on International Classification of Diseases (ICD) codes, including both ICD‐9 and ICD‐10 (Supporting Table S1); (2) patients with non‐first‐time ICU admissions were excluded; (3) patients with multiple ICU admissions were excluded; (4) patients with ICU stays of less than 24 h were excluded; and (5) patients with missing lactate or serum albumin data were excluded (Figure 1).

### 2.3. Data Extraction

Data were extracted from the MIMIC‐IV database using Navicat Premium software (version 16) through Structured Query Language queries. Variables collected within the first 24 h of ICU admission included demographic characteristics (age, sex, and race), vital signs (oxygen saturation, temperature, respiratory rate, heart rate, and blood pressure), and laboratory measurements, such as anion gap, total calcium, chloride, glucose, potassium, sodium, hemoglobin, red blood cell (RBC), white blood cell (WBC), alanine aminotransferase (ALT), aspartate aminotransferase (AST), creatinine, blood urea nitrogen (BUN), lactate, and albumin. We also documented therapeutic interventions, including mechanical ventilation, continuous renal replacement therapy (CRRT), and the administration of insulin, glucocorticoids, and vasopressors, as well as comorbidities, such as hypertension, chronic kidney disease (CKD), HLD, MI, coronary heart disease (CHD), HF, and cerebrovascular accident (CVA). Clinical severity was assessed using standardized scoring systems, including the Glasgow coma scale (GCS), Charlson score, systemic inflammatory response syndrome (SIRS) score, sequential organ failure assessment (SOFA) score, and Oxford acute severity of illness score (OASIS).

LAR was calculated using the following formula: LAR = lactate (mmol/L)/albumin (g/dL). In this study, the ratio was expressed as a variable without a specific unit.

### 2.4. Study Outcomes

The primary outcome measures were ICU mortality, defined as the proportion of patients who died during their stay in the ICU, and in‐hospital mortality, defined as the proportion of patients who died during the entire hospitalization period.

Secondary outcomes included 30‐day, 90‐day, and 365‐day mortality following ICU admission.

### 2.5. Feature Selection

Before investigating the association between LAR and ICU mortality, we adopted a two‐stage feature selection strategy combining the Boruta algorithm and SHAP analysis. The random forest‐based Boruta algorithm identifies statistically significant features by comparing their importance with randomized shadow features, iteratively classifying variables as “important” or “unimportant” to reduce dimensionality by removing redundant/irrelevant predictors [24–26]. Boruta‐selected features were then analyzed via SHAP, which quantifies each variable’s marginal contribution to model predictions using Shapley values (cooperative game theory), providing global importance rankings and intuitive visualizations to enhance model interpretability [27–29]. These complementary approaches ensured transparent and robust covariate selection for subsequent regression models.

### 2.6. Statistical Analysis

The normality of continuous variables was assessed using the Shapiro–Wilk test. Variables with a normal distribution were expressed as the mean ± standard deviation (SD) and compared using *t*‐tests, whereas variables with a non‐normal distribution were expressed as the median and interquartile range (IQR) and compared using the Kruskal–Wallis test. Categorical variables were presented as counts (percentages) and compared using Fisher’s exact test or chi‐square test as appropriate. For missing data management, variables with missing rates below 40% were handled using multiple imputation with the “mice” package in R [30] and were converted to dummy variables in the model to reduce potential estimation bias. This 40% threshold was selected in accordance with established methodological recommendations, noting that when key variables have over 40% missing data, results should be seen as hypothesis‐generating, not definitive. Using methods like multiple imputation in these cases can falsely suggest greater precision than the data supports [31]. Accordingly, Variables with missing rates exceeding 40% were excluded from the analysis (Supporting Table S2).

**FIGURE 3:**
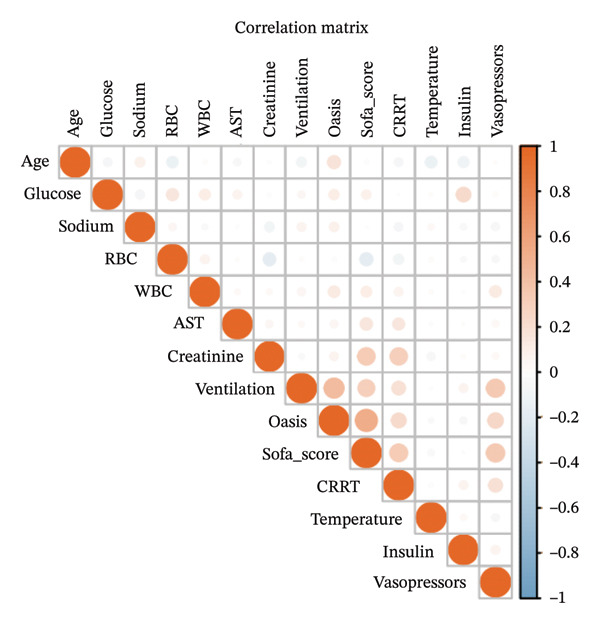
Variable correlation coefficient matrix. The matrix displays the pairwise correlations between clinical variables. Circle colors indicate the direction of correlation (orange for positive, blue for negative), while circle sizes represent the strength of the correlation (larger circles indicate absolute coefficients closer to 1). WBC, white blood cell; RBC, red blood cell; AST, aspartate aminotransferase; OASIS, Oxford acute severity of illness score; SOFA score, sequential organ failure assessment score; CRRT, continuous renal replacement therapy.

LAR was analyzed as both a continuous variable and a categorical variable. For the categorical analysis, patients were stratified into tertiles based on their LAR values. This stratification strategy was employed to ensure adequate and balanced sample sizes within each subgroup and to facilitate the assessment of potential nonlinear relationships between LAR levels and the risk of ICU mortality. The associations between LAR and mortality outcomes were evaluated using logistic regression models for ICU and in‐hospital mortality (reporting odds ratios [OR] with 95% CI) and Cox proportional hazards models for 30‐day, 90‐day, and 365‐day mortality (reporting hazard ratios [HRs] with 95% CI). We calculated Harrell’s concordance index for each fully adjusted Cox model. Three adjustment models were constructed: Model 1 (unadjusted); Model 2 (adjusted for age, sex, OASIS, and SOFA score); and Model 3 (adjusted for variables identified through Boruta and SHAP algorithms, including age, CRRT, vasopressor, OASIS, SOFA score, mechanical ventilation, insulin, creatinine, AST, glucose, temperature, sodium, RBC, and WBC), as shown in Figure 2. Correlation and multicollinearity were assessed among adjusted variables, confirming correlation coefficients < 0.6 (Figure 3) and variance inflation factors < 2 (Supporting Table S3). To further explore the temporal dynamics of the association between LAR and mortality, we performed a time‐stratified (piecewise) Cox regression model as a sensitivity analysis. The 30‐day follow‐up was divided at day 7, distinguishing an early phase (0–7 days) and a late phase (> 7 days), and time‐varying HRs for LAR were estimated within each interval.

FIGURE 2Machine learning variable screening analysis. (a) Feature selection analysis based on the Boruta algorithm. The horizontal axis shows clinical variables, while the vertical axis represents importance scores (*Z* values). The box plots demonstrate the distribution of *Z* values for variables across multiple iterations. Purple, blue, and green box plots represent the reference variables shadowMin, shadowMean, and shadowMax generated by the algorithm, establishing evaluation baselines. Red box plots indicate important variables with *Z* values significantly higher than shadowMax. Results show that variables such as LAR, SOFA score, and CRRT are significantly correlated with ICU mortality. (b) SHAP analysis of the random forest model. This displays the impact of various features on ICU mortality risk prediction, where the horizontal axis SHAP values indicate the degree and direction of influence (positive values increase mortality risk, negative values decrease it). Each point represents a patient, with colors ranging from yellow to purple indicating low to high feature values. SHAP, Shapley additive explanations; BMI, body mass index; NBPD, non‐invasive blood pressure systolic; NBPS, non‐invasive blood pressure diastolic; RR, respiratory rate; WBC, white blood cell; RBC, red blood cell; ALT, alanine aminotransferase; AST, aspartate aminotransferase; BUN, blood urea nitrogen; LAR, lactate‐to‐albumin ratio; OASIS, Oxford acute severity of illness score; GCS, Glasgow coma scale score; SOFA score, sequential organ failure assessment score; CRRT, continuous renal replacement therapy; ICU, intensive care unit.

**Figure:**
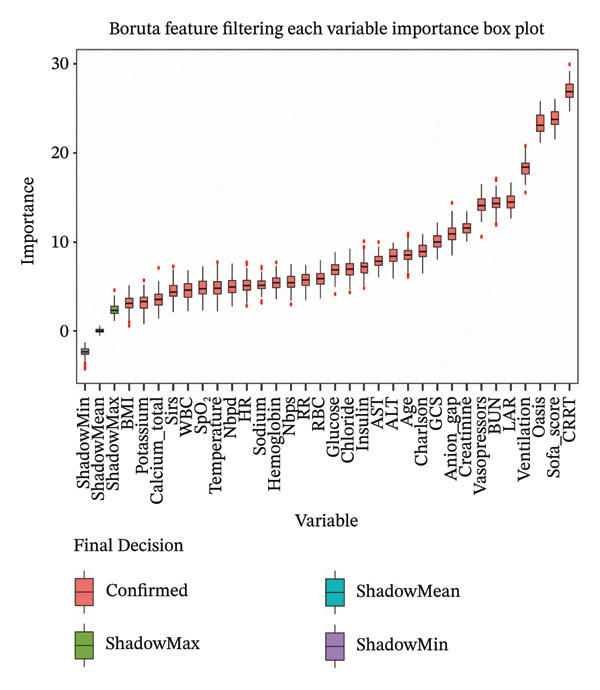
(a)

**Figure:**
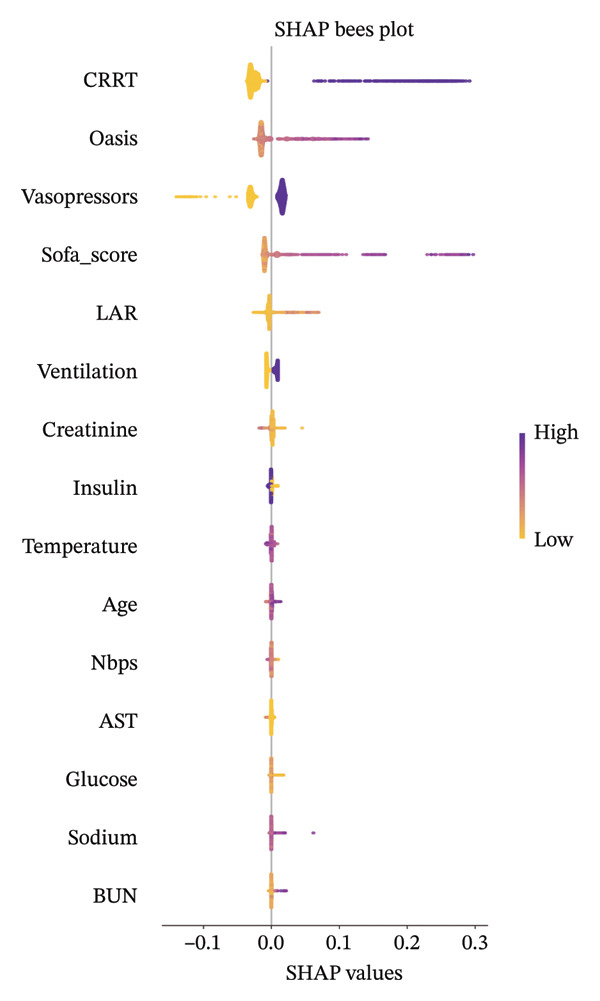
(b)

The continuous relationship between LAR and mortality outcomes was examined using RCS analysis. Additionally, threshold effects were evaluated using generalized additive models to enhance clinical applicability. Kaplan–Meier survival analysis was conducted to evaluate cumulative all‐cause mortality rates among T2DM patients at 30‐day, 90‐day, and 365‐day following ICU admission, comparing mortality across LAR tertiles.

To test the robustness of our findings, we performed sensitivity analyses using three progressive models. Model 1 remained unadjusted; Model 2 was adjusted for age, sex, race, and body mass index (BMI); and Model 3 incorporated all covariates. The prognostic value of LAR was further examined through comprehensive subgroup analyses, which were stratified by demographic characteristics, including age (≤ 60 vs. > 60 years), sex (female vs. male), race (White vs. non‐White), and BMI (≤ 25 vs. > 25 kg/m^2^). We also conducted analyses across various comorbidity subgroups, including hypertension, CKD, HLD, MI, CHD, HF, and CVA. For each subgroup analysis, both *p* values and interaction terms were calculated to assess the consistency of the prognostic value of LAR across different patient populations.

All statistical analyses were performed using SPSS (version 29), R software (version 4.2.1), and Python (version 3.10.6). Statistical significance was set at a two‐tailed *p* value < 0.05.

## 3. Results

### 3.1. Baseline Characteristics

Among 5463 critically ill T2DM patients included in this study, the median age was 70 years, with males comprising 59.9% and White patients accounting for 57.0% of the cohort. Patients were stratified into tertiles based on LAR values (T1: LAR ≤ 0.469; T2: 0.469 < LAR ≤ 0.813; T3: LAR > 0.813) for baseline characteristic comparison. The median (IQR) LAR values were 0.34 (0.29, 0.41) for T1, 0.61 (0.54, 0.69) for T2, and 1.25 (0.97, 1.79) for T3. Significant differences across LAR tertiles were observed in age and BMI, while sex and race distributions showed no statistical differences. Vital signs showed progressive changes across LAR tertiles, with increasing heart rate and respiratory rate, but decreasing systolic and diastolic blood pressure as LAR increased. Laboratory findings revealed that the T3 group, compared to T1, had significantly higher levels of blood glucose, WBC, and transaminases, while albumin levels were markedly lower. Disease severity scores, including SOFA score and OASIS, were notably higher in the T3 group. Major comorbidities included HLD, CHD, and HF. The T3 group had significantly higher proportions of patients receiving mechanical ventilation, CRRT, and vasopressor support compared to the T1 group (Supporting Table S4).

In‐hospital and ICU‐related mortality among different groups are shown in Supporting Table S5.

### 3.2. Association Between LAR and Primary Outcomes

Multivariable logistic regression analysis demonstrated significant associations between LAR and both ICU and in‐hospital mortality (Table 1). When analyzing LAR as a continuous variable in the unadjusted Model 1, significant associations were found with ICU mortality and in‐hospital mortality. These associations persisted, though attenuated, in the adjusted Models 2 and Model 3. In tertile‐based analyses, compared to T1 (lowest tertile), T3 showed significantly higher risks in the unadjusted Model 1 for both ICU mortality and in‐hospital mortality. These associations remained significant in the fully adjusted Model 3: ICU mortality and in‐hospital mortality, with significant dose–response relationships.

**TABLE 1:** Associations between LAR and mortality outcomes in patients with T2DM in ICU.

	Model 1			Model 2			Model 3		
	HR/OR (95% CI)	*p* value	*p* for trend	HR/OR (95% CI)	*p* value	*p* for trend	HR/OR (95% CI)	*p* value	*p* for trend
ICU mortality									
LAR (continuous)	1.64 (1.52, 1.78)	< 0.001		1.21 (1.11, 1.32)	< 0.001		1.18 (1.07, 1.30)	< 0.001	
T1	Reference		< 0.001	Reference		< 0.001	Reference		0.002
T2	1.50 (1.20, 1.89)	< 0.001		1.27 (1.00, 1.60)	0.047		1.20 (0.94, 1.53)	0.142	
T3	2.84 (2.31, 3.50)	< 0.001		1.56 (1.24, 1.95)	< 0.001		1.45 (1.14, 1.85)	0.003	
In‐hospital mortality									
LAR (continuous)	1.62 (1.50, 1.75)	< 0.001		1.27 (1.17, 1.38)	< 0.001		1.25 (1.14, 1.36)	< 0.001	
T1	Reference		< 0.001	Reference		< 0.001	Reference		< 0.001
T2	1.50 (1.24, 1.81)	< 0.001		1.31 (1.08, 1.59)	0.007		1.26 (1.03, 1.54)	0.025	
T3	2.71 (2.27, 3.24)	< 0.001		1.73 (1.43, 2.10)	< 0.001		1.66 (1.36, 2.04)	< 0.001	
30‐day ICU mortality									
LAR (continuous)	1.38 (1.32, 1.43)	< 0.001		1.16 (1.11, 1.22)	< 0.001		1.15 (1.09, 1.21)	< 0.001	
T1	Reference		< 0.001	Reference		< 0.001	Reference		< 0.001
T2	1.51 (1.29, 1.76)	< 0.001		1.32 (1.13, 1.55)	0.007		1.29 (1.10, 1.52)	0.002	
T3	2.42 (2.09, 2.80)	< 0.001		1.61 (1.38, 1.88)	< 0.001		1.56 (1.33, 1.83)	< 0.001	
90‐day ICU mortality									
LAR (continuous)	1.34 (1.29, 1.39)	< 0.001		1.16 (1.11, 1.21)	< 0.001		1.15 (1.10, 1.21)	< 0.001	
T1	Reference		< 0.001	Reference		< 0.001	Reference		< 0.001
T2	1.37 (1.21, 1.56)	< 0.001		1.23 (1.08, 1.40)	0.002		1.22 (1.07, 1.39)	0.002	
T3	2.04 (1.81, 2.30)	< 0.001		1.47 (1.30, 1.67)	< 0.001		1.47 (1.29, 1.67)	< 0.001	
365‐day ICU mortality									
LAR (continuous)	1.28 (1.23, 1.33)	< 0.001		1.13 (1.08, 1.18)	< 0.001		1.14 (1.09, 1.19)	< 0.001	
T1	Reference		< 0.001	Reference		< 0.001	Reference		< 0.001
T2	1.32 (1.19, 1.47)	< 0.001		1.20 (1.08, 1.33)	< 0.001		1.21 (1.08, 1.35)	0.002	
T3	1.72 (1.55, 1.91)	< 0.001		1.31 (1.18, 1.46)	< 0.001		1.35 (1.21, 1.51)	< 0.001	

*Note:* T1: LAR ≤ 0.469; T2: 0.469 < LAR ≤ 0.813; T3: LAR > 0.813. Model 1: Unadjusted for any confounders; Model 2: Adjusted for age, temperature, SOFA score, and OASIS; Model 3: Based on Model 2, further adjusted for glucose, sodium, aspartate aminotransferase, creatinine, ventilation, continuous renal replacement therapy, insulin, vasopressor, white blood cell; and red blood cell.

Abbreviations: HR, hazard ratio; ICU, intensive care unit; LAR, lactate‐to‐albumin ratio; OR, odds ratio; T2DM, Type 2 diabetes mellitus.

RCS analysis revealed a linear relationship between LAR and ICU mortality (Figure 4(a)), but a nonlinear relationship with in‐hospital mortality (Figure 4(b)). Further investigation of the threshold effect between LAR and in‐hospital mortality identified a critical threshold of 2.10 (Table 2). Below this threshold, each unit increase in LAR was associated with mortality risk becoming 2.01 times. However, above the threshold (LAR > 2.10), this association became nonsignificant. The log‐likelihood ratio test supported the threshold effect model over the linear model. Based on the optimal cutoff value of LAR, 5122 patients were classified into the LAR < 2.10 group, among whom 848 experienced in‐hospital death; 341 patients were included in the LAR ≥ 2.10 group, with 137 in‐hospital deaths (Supporting Table S6). Our analysis revealed that the data were mainly concentrated in the LAR < 2.10 interval, which covered the vast majority of the study population, while the LAR ≥ 2.10 interval was the high‐value tail of the data distribution with a relatively limited sample size.

FIGURE 4Restricted cubic spline function between LAR and ICU mortality (a) and hospital mortality (b), adjusted for age, CRRT, vasopressor, OASIS, SOFA score, mechanical ventilation, insulin, creatinine, AST, glucose, temperature, sodium, RBC, and WBC. LAR, lactate‐to‐albumin ratio.

**Figure:**
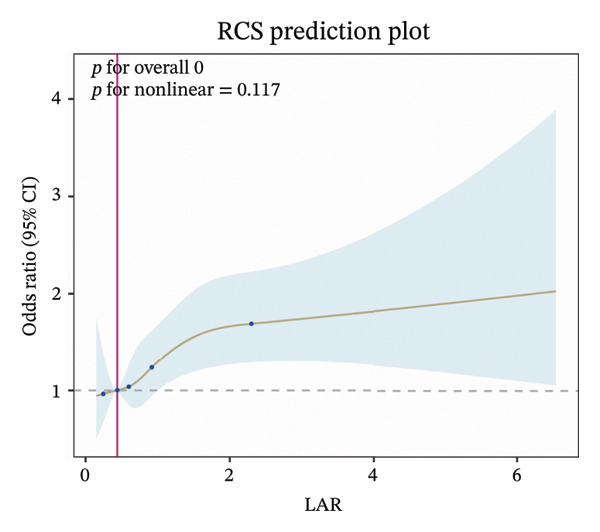
(a)

**Figure:**
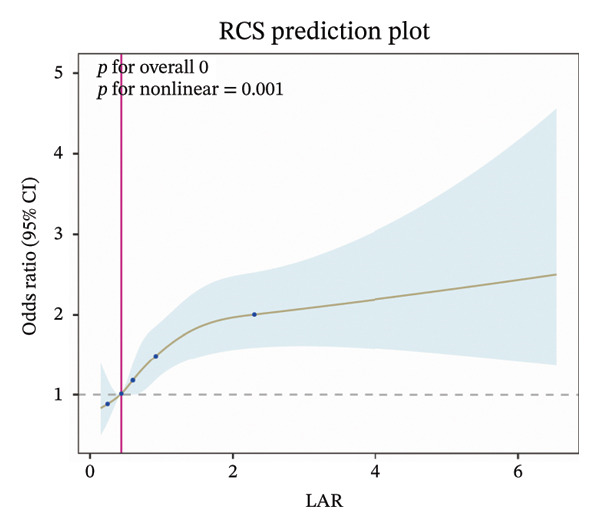
(b)

**TABLE 2:** Threshold effect.

	Row name	Value	*p* value
LAR	Model 1 Line Effect	1.37 (95% CI: 1.25–1.50)	< 0.001
LAR	Model 2 Threshold(W)	2.10	
LAR	Model 2 < W Effect	2.01 (95% CI: 1.73–2.33)	< 0.001
LAR	Model 2 > W Effect	0.93 (95% CI: 0.80–1.07)	0.308
LAR	Log‐likelihood ratio test		< 0.001

### 3.3. Association Between LAR and Secondary Outcomes

We calculated Harrell’s concordance index for each fully adjusted Cox model. All three models exhibited acceptable to good discriminative performance, with the corresponding results summarized in Supporting Table S7. The proportional hazards assumption was assessed using the Schoenfeld residual test. Global tests for all three models yielded statistically significant results (30‐day: *p* = 1.17 × 10^−23^; 90‐day: *p* = 1.49 × 10^−41^; 365‐day: *p* = 2.38 × 10^−58^). Given the large sample size in the present study (*N* = 5463), such findings were not unexpected, as the Schoenfeld test is highly sensitive to minor violations of the PH assumption in large datasets, which usually carry limited clinical relevance. We therefore visually inspected the scaled Schoenfeld residuals of LAR at the three follow‐up time points. The residuals were randomly distributed around the zero line with no obvious directional trend, supporting that the PH assumption was reasonably met in practice. Thus, the reported HRs can be interpreted as time‐averaged estimates over the follow‐up interval.

Cox proportional hazards regression analysis demonstrated significant associations between LAR and both short‐term and long‐term outcomes in ICU patients (Table 1). When LAR was analyzed as a continuous variable in the fully adjusted Model 3, each unit increase in LAR was associated with significantly higher risks of 30‐day and 365‐day mortality. In tertile‐based analyses, compared with T1, patients in T3 exhibited significantly increased mortality risks in Model 3 for 30‐day, 90‐day, and 365‐day mortality. A significant dose–response relationship was observed at all time points, indicating a strong association between LAR levels and both short‐term and long‐term survival outcomes in ICU patients.

The time‐stratified sensitivity analysis demonstrated a time‐dependent effect of LAR on 30‐day mortality. In the early phase (0–7 days), elevated LAR remained a strong and independent predictor of mortality (HR = 1.221, 95% CI: 1.149–1.298, *p* < 0.001). In contrast, this association was markedly attenuated during the late phase (> 7 days: HR = 1.056, 95% CI: 0.976–1.142, *p* = 0.176).

Further analysis of the relationship between LAR and survival outcomes was conducted using RCS analysis and Kaplan–Meier survival analysis. The RCS analysis, after adjusting for machine learning‐selected covariates, revealed a significant nonlinear relationship between LAR and survival outcomes in T2DM patients (*p* for nonlinear < 0.001) (Supporting Figure 1). Kaplan–Meier survival curve analysis demonstrated significant differences among the three groups (Log‐rank test, *p* < 0.001), with higher LAR levels associated with lower survival rates (Supporting Figure 2).

### 3.4. Sensitivity Analysis

Sensitivity analyses (Table 3) confirmed the stability of associations between LAR and outcomes in T2DM patients after full adjustment (Model 3). As a continuous variable, LAR maintained significant associations with ICU mortality and in‐hospital mortality. Compared with T1, patients in T3 exhibited significantly higher risks of ICU mortality and in‐hospital mortality. Cox regression analysis similarly demonstrated significant associations between LAR and long‐term outcomes. In Model 3, each unit increase in LAR was associated with increased risks of 30‐day, 90‐day, and 365‐day mortality. Compared with T1, T3 showed significantly higher mortality risks across all time points, with significant dose–response relationships. These findings were consistent with the primary analysis, further supporting the robust association between LAR and outcomes in ICU patients with T2DM.

**TABLE 3:** Sensitivity analysis of associations between LAR and mortality outcomes in patients with T2DM in ICU.

	Model 1			Model 2			Model 3		
	HR/OR (95% CI)	*p* value	*p* for trend	HR/OR (95% CI)	*p* value	*p* for trend	HR/OR (95% CI)	*p* value	*p* for trend
ICU mortality									
LAR (continuous)	1.64 (1.52, 1.78)	< 0.001		1.16 (1.53, 1.80)	< 0.001		1.19 (1.07, 1.34)	0.002	
T1	Reference		< 0.001	Reference		< 0.001	Reference		0.004
T2	1.50 (1.20, 1.89)	< 0.001		1.48 (1.18, 1.86)	< 0.001		1.15 (0.90, 1.48)	0.261	
T3	2.84 (2.31, 3.50)	< 0.001		2.82 (2.28, 3.48)	< 0.001		1.45 (1.12, 1.88)	0.004	
In‐hospital mortality									
LAR (continuous)	1.62 (1.50, 1.75)	< 0.001		1.62 (1.50, 1.75)	< 0.001		1.24 (1.12, 1.37)	< 0.001	
T1	Reference		< 0.001	Reference		< 0.001	Reference		< 0.001
T2	1.50 (1.24, 1.81)	< 0.001		1.46 (1.21, 1.77)	< 0.001		1.22 (0.99, 1.50)	0.064	
T3	2.71 (2.27, 3.24)	< 0.001		2.66 (2.22, 3.18)	< 0.001		1.66 (1.34, 2.06)	< 0.001	
30‐day ICU mortality									
LAR (continuous)	1.38 (1.32, 1.43)	< 0.001		1.38 (1.32, 1.44)	< 0.001		1.14 (1.07, 1.22)	< 0.001	
T1	Reference		< 0.001	Reference		< 0.001	Reference		< 0.001
T2	1.51 (1.29, 1.76)	< 0.001		1.46 (1.25, 1.71)	< 0.001		1.23 (1.05, 1.45)	0.010	
T3	2.42 (2.09, 2.80)	< 0.001		2.33 (2.01, 2.70)	< 0.001		1.53 (1.29, 1.80)	< 0.001	
90‐day ICU mortality									
LAR (continuous)	1.34 (1.29, 1.39)	< 0.001		1.34 (1.29, 1.39)	< 0.001		1.15 (1.10, 1.23)	< 0.001	
T1	Reference		< 0.001	Reference		< 0.001	Reference		< 0.001
T2	1.37 (1.21, 1.56)	< 0.001		1.33 (1.17, 1.51)	< 0.001		1.19 (1.04, 1.36)	0.009	
T3	2.04 (1.81, 2.30)	< 0.001		1.96 (1.73, 2.21)	< 0.001		1.47 (1.28, 1.69)	< 0.001	
365‐day ICU mortality									
LAR (continuous)	1.28 (1.23, 1.33)	< 0.001		1.27 (1.23, 1.32)	< 0.001		1.15 (1.09, 1.21)	< 0.001	
T1	Reference		< 0.001	Reference		< 0.001	Reference		< 0.001
T2	1.32 (1.19, 1.47)	< 0.001		1.28 (1.15, 1.42)	< 0.001		1.19 (1.07, 1.33)	0.002	
T3	1.72 (1.55, 1.91)	< 0.001		1.65 (1.49, 1.83)	< 0.001		1.36 (1.21, 1.53)	< 0.001	

*Note:* T1: LAR ≤ 0.469; T2: 0.469 < LAR ≤ 0.813; T3: LAR > 0.813. Model 1: Unadjusted for any confounders. Model 2: Adjusted for age, sex, race, and body mass index. Model 3: Based on Model 2, further adjusted for anion gap, calcium total, chloride, glucose, potassium, sodium, hemoglobin, white blood cell, red blood cell, alanine aminotransferase, aspartate aminotransferase, creatinine, blood urea nitrogen, hypertension, chronic kidney disease, hyperlipidemia, coronary heart disease, myocardial infarct, heart failure, cerebral vascular accident, mechanical ventilation, Glasgow coma scale score, Charlson, sirs, oxford acute severity of illness score, sequential organ failure assessment score, continuous renal replacement therapy, SpO_2_, temperature, diastolic blood pressure, systolic blood pressure, heart rate, respiratory rate, insulin, glucocorticoids, and vasopressor.

Abbreviations: HR, hazard ratio; ICU, intensive care unit; LAR, lactate‐to‐albumin ratio; OR, odds ratio.

### 3.5. Subgroup Analysis

Subgroup analyses (Figure 5) demonstrated consistent associations between LAR and both ICU and in‐hospital mortality across most subgroups. Overall, LAR showed significant associations with ICU mortality and in‐hospital mortality. Significant interactions were observed in gender subgroups for ICU mortality and HLD subgroups for in‐hospital mortality. Specifically, the association between LAR and ICU mortality was more pronounced in male patients, while the association with in‐hospital mortality was stronger in patients without HLD. No significant interactions were observed in other subgroups. Additionally, subgroup analyses for 30‐day, 90‐day, and 365‐day mortality showed no significant interactions (Supporting Figure 3).

FIGURE 5Subgroup analyses for the association of LAR with ICU mortality (a) and hospital mortality (b), adjusted for age, CRRT, vasopressor, OASIS, SOFA score, mechanical ventilation, insulin, creatinine, AST, glucose, temperature, sodium, RBC, and WBC. OR, odds ratio, LAR, lactate‐to‐albumin ratio, ICU, intensive care unit, BMI, body mass index, HTN, hypertension, CKD, chronic kidney disease, HLD, hyperlipidemia, MI, myocardial infarction, CHD, coronary heart disease, HF, heart failure, CVA, cerebrovascular accident.

**Figure:**
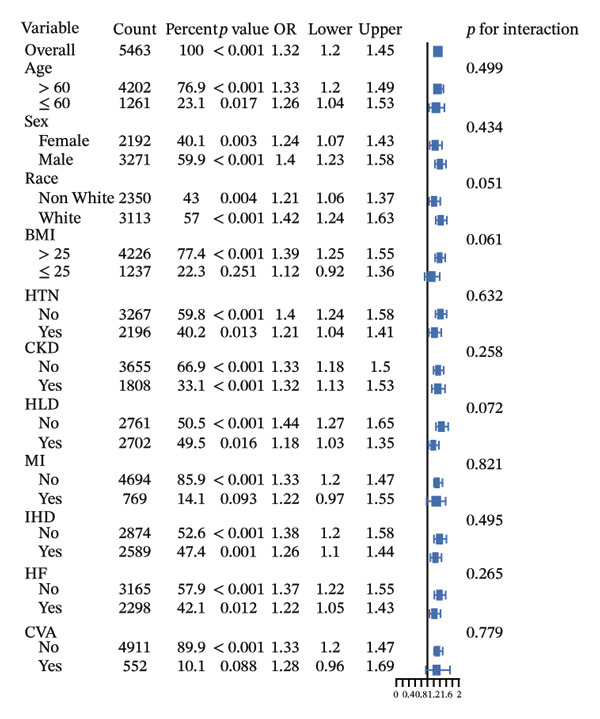
(a)

**Figure:**
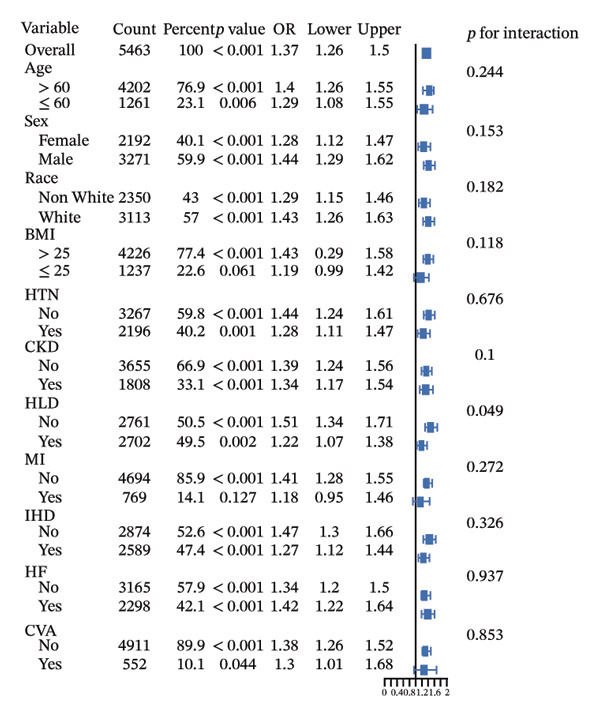
(b)

## 4. Discussion

In this study, we employed the publicly accessible MIMIC‐IV 3.0 database to explore, for the first time, the association between the LAR and adverse outcomes in critically ill patients with T2DM. The findings indicated that an elevated LAR ratio was significantly correlated with increased mortality rates in the ICU, as well as in‐hospital and 30‐day, 90‐day, and 365‐day mortality among critically ill T2DM patients. This significant association remained robust even after adjusting for multiple confounding variables. Sensitivity analyses, conducted with various adjusted covariates, consistently supported these results. Subgroup analyses identified an interaction between gender and ICU mortality, as well as between HLD subgroups and in‐hospital mortality.

In recent years, the LAR has been proposed as a potential prognostic biomarker for various critical conditions, such as the return of spontaneous circulation after out‐of‐hospital cardiac arrest, acute kidney injury, and gastrointestinal bleeding [32–34]. Numerous clinical studies have confirmed that elevated LAR is associated with poor outcomes in severe infectious and cardiovascular diseases, including community‐acquired pneumonia, sepsis, CHD, and AMI [22, 35–37]. The present study is the first to specifically evaluate the prognostic value of LAR in critically ill patients with T2DM. Our results demonstrate that LAR is not only an independent predictor of ICU and in‐hospital mortality in this population, but also exhibits strong predictive power for 30‐day, 90‐day, and 365‐day ICU mortality. Notably, the incidence and mortality of infectious and cardiovascular diseases are significantly higher in T2DM patients than in non‐T2DM patients [38, 39]. Consequently, our findings are consistent with previous studies and further endorse the broader applicability of LAR as a prognostic tool for critically ill patients.

Lactate serves as a sensitive indicator of tissue hypoperfusion and metabolic dysregulation [40]. Studies have demonstrated that hyperlactatemia is significantly correlated with increased morbidity and mortality among critically ill patients [41, 42]. In research concerning T2DM, Crawford and Cox et al. observed markedly elevated plasma lactate levels in individuals with T2DM and diabetic ketoacidosis [11, 43]. This elevation may be attributed to impaired oxidative capacity and exacerbated insulin resistance. Mitochondrial oxidative phosphorylation dysfunction in T2DM patients contributes to hypoxic states, promoting lactate accumulation [44]. Hypoxia not only impairs glucose tolerance by increasing oxidative stress and lipid peroxidation but also aggravates insulin resistance by activating the sympathetic nervous system and releasing catabolic hormones, thereby accelerating T2DM progression [45]. Serum albumin, a multifunctional carrier protein synthesized in the liver, serves as an important clinical indicator of nutritional status and inflammation levels [46, 47]. Studies have shown an inverse relationship between serum albumin levels and T2DM risk [48]. Low albumin levels are strong predictors of increased all‐cause and cardiovascular mortality in hospitalized patients [49]. In critical care settings, hypoalbuminemia is closely associated with prolonged ICU stays and poor outcomes.^35^ In T2DM patients, serum albumin levels can be reduced by several mechanisms, including increased protein catabolism [50], chronic low‐grade inflammation that impairs hepatic albumin synthesis [51, 52], and proteinuria secondary to diabetic nephropathy [53]. Reduced albumin levels lead to malnutrition, weakened immunity, and higher organ failure risk. In short, a high LAR in critically ill T2DM patients signals greater insulin resistance, severe microcirculatory issues, and nutritional imbalances, resulting in negative outcomes.

We used the Boruta algorithm and SHAP methods for feature selection, which are popular for simplifying models and enhancing interpretability in multivariate feature screening. LAR emerged as nearly as important as the SOFA score and OASIS for predicting mortality risk in T2DM patients. Sensitivity analyses confirmed the stability of these algorithms. Importantly, we discovered a significant nonlinear relationship and threshold effect between LAR and in‐hospital mortality risk in T2DM patients, which has crucial clinical implications. A threshold effect analysis identified a critical LAR value of 2.10. Below this threshold, each unit increase in LAR nearly doubled the mortality risk, highlighting LAR as a key prognostic indicator. However, when LAR exceeded 2.10, the association between LAR and mortality risk diminished, suggesting that patients with LAR ≥ 2.10 are already at high risk, and the predictive performance of LAR plateaued. Most data points fall below LAR 2.10, indicating potential data dependence and possible fluctuations when applied to other populations. Despite this, LAR remains valuable for early risk stratification of in‐hospital mortality in patients with T2DM, aiding in early identification of high‐risk individuals and optimizing medical resource allocation.

The time‐stratified sensitivity analysis showed that the effect of LAR on 30‐day mortality changes over time, which is biologically plausible. In the early phase of critical illness, hypoalbuminemia and elevated lactate indicate severe inflammation and tissue hypoperfusion, respectively, and are most significant in the first week of ICU admission. After 7 days, survival bias and treatments may reduce the prognostic value of LAR. These findings suggest that LAR is a useful early warning biomarker, emphasizing the need for dynamic monitoring of LAR during the first week in ICU patients with T2DM.

Subgroup analysis revealed a notable gender difference: the link between LAR and ICU mortality was stronger in males. This disparity might be due to smaller sample sizes reducing effect sizes or gender‐specific metabolic responses to stress in diabetic patients. Prior research suggests that critically ill male patients often show greater metabolic disturbances and inflammatory responses [54, 55]. The stronger predictive power of LAR in male patients highlights the need for gender‐specific monitoring and intervention strategies in clinical practice. Additionally, HLD status moderates the predictive value of LAR, with a stronger association between LAR and in‐hospital mortality in patients without HLD, suggesting hyperlipidemia influences metabolic states [56]. It underscores the importance of incorporating patients’ baseline metabolic status into prognostic evaluation.

### 4.1. Limitation

While progress has been made in investigating the association between LAR and mortality in critically ill patients with T2DM, this study has several limitations. The study only enrolled ICU patients, which restricts the generalizability to non‐ICU patients with T2DM; future studies should include broader populations to confirm these findings. Additionally, although major confounders were adjusted for, unmeasured confounders may still influence the results. As a retrospective cohort study, causal inference is limited, supporting the necessity for large‐scale, multicenter prospective investigations. Further research should also explore the clinical applications of LAR in T2DM and other metabolic disorders to improve patient care.

## 5. Conclusion

LAR is associated with increased mortality in critically ill patients with comorbid T2DM and may serve as an early prognostic biomarker, but large‐scale studies needed to validate these findings and its clinical utility.

**Nomenclature**
T2DM: Type 2 diabetes mellitus
LAR: Lactate‐to‐albumin ratio
MIMIC‐IV: Medical information mart for intensive care IV
ICU: Intensive care unit
RCS: Restricted cubic spline
SHAP: SHapley Additive exPlanations
HLD: Hyperlipidemia
CITI: Collaborative institutional training initiative
ICD: International classification of diseases
RBC: Red blood cell
WBC: White blood cell
ALT: Alanine aminotransferase
AST: Aspartate aminotransferase
BUN: Blood urea nitrogen
CRRT: Continuous renal replacement therapy
CKD: Chronic kidney disease
MI: Myocardial infarction
CHD: Coronary heart disease
HF: Heart failure
CVA: Cerebrovascular accident
GCS: Glasgow coma scale
SIRS: Systemic inflammatory response syndrome
OASIS: Oxford acute severity of illness score
SOFA: Sequential organ failure assessment
OR: Odds ratios
HRs: Hazard ratios

## Author Contributions

Yang Liu and Zhi‐Jun Li contributed to the study concept and study design. Zhuo‐Ting Cheng and Jia‐Liang Wang performed the statistical analysis and data interpretation. Yan‐Bo Zhao and Zheng‐Ming Zhao were responsible for the quality control of the data. Zhuo‐Ting Cheng, Jia‐Liang Wang, and Yang Liu performed the literature research and data extraction. Yang Liu obtained the CITI certification and the necessary permissions to use the MIMIC‐IV database. All authors contributed to the writing of the manuscript.

## Funding

This study was supported by Hubei Provincial Natural Science Foundation of China (Grant No. 2024AFB390) and the Shiyan City Guiding Research Project (Grant No. 24Y115).

## Disclosure

All authors approved the final manuscript and concur with the submission.

## Ethics Statement

The MIMIC‐IV database was accessed under credential number 65970781, with data being de‐identified and publicly available, thereby exempting the need for additional ethical approval or informed consent.

## Conflicts of Interest

The authors declare no conflicts of interest.

## Supporting Information

Additional supporting information can be found online in the Supporting Information section.

## Supporting information


**Supporting Information 1** Supporting Table S1 Inclusion criteria.

**Supporting Information 2** Supporting Table S2 Missing values proportions.

**Supporting Information 3** Supporting Table S3 Variance inflation factor.

**Supporting Information 4** Supporting Table S4 Baseline characteristics of critically ill patients with T2DM in ICU.

**Supporting Information 5** Supporting Table S5 Comparison of in‐hospital and ICU‐related mortality among different groups.

**Supporting Information 6** Supporting Table S6 In‐hospital mortality by LAR Cut‐off Value.

**Supporting Information 7** Supporting Table S7 Harrell’s C‐index of the prognostic model across different follow‐up periods.

**Supporting Information 8** Supporting Figure 1 Restricted cubic spline function between LAR and 30‐day ICU mortality (a), 90‐day ICU mortality (b), and 365‐day ICU mortality (c), adjusted for age, CRRT, vasopressor, OASIS, SOFA score, mechanical ventilation, insulin, creatinine, AST, glucose, temperature, sodium, RBC, and WBC. LAR, lactate‐to‐albumin ratio.

**Supporting Information 9** Supporting Figure 2 Kaplan–Meier survival curves for 30‐day (a), 90‐day (b), and 365‐day (c) all‐cause mortality according to tertiles of LAR.

**Supporting Information 10** Supporting Figure 3 Subgroup analyses for the association of LAR with 30‐day ICU mortality (a), 90‐day ICU mortality (b), and 365‐day ICU mortality (c), adjusted for age, CRRT, vasopressor, OASIS, SOFA score, mechanical ventilation, insulin, creatinine, AST, glucose, temperature, sodium, RBC, and WBC. HR: Hazards ratio; LAR: lactate‐to‐albumin ratio; ICU: intensive care unit, BMI, body mass index, HTN, hypertension, CKD, chronic kidney disease, HLD, hyperlipidemia, MI, myocardial infarction, CHD, coronary heart disease, HF, heart failure, CVA, cerebrovascular accident.

## Data Availability

The datasets generated and analyzed during the current study are available in the MIMIC‐IV database (https://mimic.mit.edu).
