# Supplementary material for: Association Between the Lactate‐to‐Albumin Ratio and ICU/In‐Hospital Mortality in Critically Ill Patients With Comorbid Type 2 Diabetes Mellitus : A Cohort Study Utilizing the MIMIC‐IV Database
Source: Emerg Med Int. 2026 Apr 13;2026:2751114. doi: 10.1155/emmi/2751114 (PMC13072064; doi:10.1155/emmi/2751114)
Supplement: Supplementary file 1 — Supporting Information 1 Supporting Table S1 Inclusion criteria. [file EMMI-2026-2751114-s001.docx]

| Supplementary Table S1 Inclusion criteria | | | |
| --- | --- | --- | --- |
|  | **Subcategory** | **ICD Code** | **Diagnosis Description** |
| T2DM Basic Diagnosis | Uncomplicated T2DM | E1121 | T2DM with complications |
|  |  | E1122 | T2DM with complications |
|  |  | E1129 | T2DM with other specified complications |
| T2DM with Renal Complications |  | E11.2x | T2DM with kidney complications |
| T2DM with Ophthalmic Complications | Retinopathy | E11.31x | T2DM with retinopathy |
|  | Cataract | E11.36 | T2DM with diabetic cataract |
|  | Other eye complications | E11.39 | T2DM with other ophthalmic complications |
| T2DM with Neurological Complications | Mononeuropathy | E11.41 | T2DM with mononeuropathy |
|  | Polyneuropathy | E11.42 | T2DM with polyneuropathy |
|  | Autonomic neuropathy | E11.43 | T2DM with autonomic neuropathy |
| T2DM with Circulatory Complications | Peripheral vascular disease | E11.51 | T2DM with peripheral vascular disease |
|  | Other circulatory complications | E11.59 | T2DM with other circulatory complications |
| Secondary diabetes |  | 24990 | Secondary diabetes and unspecified diabetes |
| Special Types of T2DM with Complications | Ketoacidosis | 25010 | T2DM with ketoacidosis |
|  |  | 25012 | T2DM with ketoacidosis, uncontrolled |
|  | Hyperosmolar state | 25020 | T2DM with hyperosmolarity |
|  |  | 25022 | T2DM with hyperosmolarity, uncontrolled |
|  | Renal complications | 25040 | T2DM with renal manifestations |
|  |  | 25042 | T2DM with renal manifestations, uncontrolled |
|  | Ophthalmic complications | 25050 | T2DM with ophthalmic manifestations |
|  |  | 25052 | T2DM with ophthalmic manifestations, uncontrolled |
|  | Neurological complications | 25060 | T2DM with neurological manifestations |
|  |  | 25062 | T2DM with neurological manifestations, uncontrolled |
|  | Peripheral circulatory disorders | 25070 | T2DM with peripheral circulatory disorders |
|  |  | 25072 | T2DM with peripheral circulatory disorders, uncontrolled |
|  | Other complications | 25080 | T2DM with other specified manifestations |
|  |  | 25082 | T2DM with other specified manifestations, uncontrolled |
|  | Unspecified complications | 25090 | T2DM with unspecified complications |
|  |  | 25092 | T2DM with unspecified complications, uncontrolled |

T2DM, Type 2 diabetes mellitus, ICD: International Classification of Diseases.
