# Supplementary material for: Association Between the Lactate‐to‐Albumin Ratio and ICU/In‐Hospital Mortality in Critically Ill Patients With Comorbid Type 2 Diabetes Mellitus : A Cohort Study Utilizing the MIMIC‐IV Database
Source: Emerg Med Int. 2026 Apr 13;2026:2751114. doi: 10.1155/emmi/2751114 (PMC13072064; doi:10.1155/emmi/2751114)
Supplement: Supplementary file 2 — Supporting Information 2 Supporting Table S2 Missing values proportions. [file EMMI-2026-2751114-s002.docx]

Supplementary Table S2 Missing values proportions of variables

| **Variable** | **Missing values, n (%)** |
| --- | --- |
| Height | 2046 (37.45) |
| Weight | 54 (0.99) |
| Systolic blood pressure | 55 (1.00) |
| Diastolic blood pressure | 55 (1.00) |
| Temperature | 51 (0.93) |
| Oxygen saturation | 1 (0.01) |
| Anion gap | 2 (0.03) |
| Calcium Total | 25 (0.46) |
| Sodium | 2 (0.03) |
| Potassium | 7 (0.13) |
| Chloride | 1 (0.01) |
| RBC | 24 (0.44) |
| WBC | 28 (0.51) |
| Glucose | 2 (0.03) |
| Hemoglobin | 22 (0.40) |
| ALT | 340 (6.22) |
| AST | 257 (4.70) |
| Creatinine | 1 (0.01) |
| BUN | 3 (0.05) |

WBC, white blood cell; RBC, red blood cell; ALT, alanine aminotransferase; AST, aspartate aminotransferase; BUN, blood urea nitrogen.
