# Supplementary material for: Association Between the Lactate‐to‐Albumin Ratio and ICU/In‐Hospital Mortality in Critically Ill Patients With Comorbid Type 2 Diabetes Mellitus : A Cohort Study Utilizing the MIMIC‐IV Database
Source: Emerg Med Int. 2026 Apr 13;2026:2751114. doi: 10.1155/emmi/2751114 (PMC13072064; doi:10.1155/emmi/2751114)
Supplement: Supplementary file 3 — Supporting Information 3 Supporting Table S3 Variance inflation factor. [file EMMI-2026-2751114-s003.docx]

Supplementary Table S3 Variance inflation factor (VIF) of model variables

| Variable |  |  |  |  | VIF |
| --- | --- | --- | --- | --- | --- |
| Age |  |  |  |  | 1.16965635910868 |
| CRRT |  |  |  |  | 1.3560889854605 |
| Vasopressors |  |  |  |  | 1.12191481448573 |
| LAR |  |  |  |  | 1.25542793046782 |
| Sofa score |  |  |  |  | 1.80239370252472 |
| OASIS |  |  |  |  | 1.59864555485251 |
| Temperature |  |  |  |  | 1.0241721909675 |
| Glucose |  |  |  |  | 1.15360436044673 |
| Sodium |  |  |  |  | 1.04338783364539 |
| AST |  |  |  |  | 1.1074962314832 |
| Creatinine |  |  |  |  | 1.32162867184364 |
| Ventilation |  |  |  |  | 1.23146826782695 |
| Insulin |  |  |  |  | 1.12259195600506 |
| WBC |  |  |  |  | 1.06731845692425 |
| RBC |  |  |  |  | 1.1273725875249 |

WBC, white blood cell; RBC, red blood cell; AST, aspartate aminotransferase; LAR, lactate to albumin ratio; OASIS, oxford acute severity of illness score; SOFA score, sequential organ failure assessment score; CRRT, continuous renal replacement therapy.
