# Supplementary material for: Association Between the Lactate‐to‐Albumin Ratio and ICU/In‐Hospital Mortality in Critically Ill Patients With Comorbid Type 2 Diabetes Mellitus : A Cohort Study Utilizing the MIMIC‐IV Database
Source: Emerg Med Int. 2026 Apr 13;2026:2751114. doi: 10.1155/emmi/2751114 (PMC13072064; doi:10.1155/emmi/2751114)
Supplement: Supplementary file 4 — Supporting Information 4 Supporting Table S4 Baseline characteristics of critically ill patients with T2DM in ICU. [file EMMI-2026-2751114-s004.docx]

Baseline characteristics across LAR tertiles groups

| **Characteristic** | **Overall** | **T1** | **T2** | **T3** | ***P*-value** |
| --- | --- | --- | --- | --- | --- |
|  | **N = 5,463** | **N = 1,828** | **N = 1,815** | **N = 1,820** |  |
| **Demographics** |  |  |  |  |  |
| Age, years | 70.00 (61.00, 79.00) | 69.00 (60.00, 78.00) | 70.00 (62.00, 78.00) | 70.00 (61.00, 79.00) | 0.019 |
| Sex, n (%) |  |  |  |  | 0.060 |
| Male | 3,271 (59.9%) | 1,094 (59.8%) | 1,122 (61.8%) | 1,055 (58.0%) |  |
| Female | 2,192 (40.1%) | 734 (40.2%) | 693 (38.2%) | 765 (42.0%) |  |
| Race, n (%) |  |  |  |  | 0.138 |
| White | 3,113 (57.0%) | 1,062 (58.1%) | 1,048 (57.7%) | 1,003 (55.1%) |  |
| Non-white | 2,350 (43.0%) | 766 (41.9%) | 767 (42.3%) | 817 (44.9%) |  |
| BMI, kg/m^2^ | 29.99 (25.53, 35.42) | 30.35 (25.94, 35.74) | 30.21 (25.59, 35.61) | 29.39 (25.11, 34.84) | <0.001 |
| **Vital sign** |  |  |  |  |  |
| Heart rate, beats/min | 88.00 (77.00, 104.00) | 85.00 (73.00, 99.00) | 87.00 (77.00, 102.00) | 93.00 (80.00, 110.00) | <0.001 |
| Respiratory rate, beats/min | 19.00 (16.00, 24.00) | 19.00 (16.00, 23.00) | 19.00 (16.00, 24.00) | 20.00 (16.00, 24.00) | <0.001 |
| SpO_2_, % | 98.00 (95.00, 100.00) | 98.00 (95.00, 100.00) | 98.00 (95.00, 100.00) | 98.00 (95.00, 100.00) | 0.238 |
| Temperature, ℉ | 98.20 (97.70, 98.80) | 98.20 (97.70, 98.80) | 98.20 (97.70, 98.80) | 98.10 (97.60, 98.80) | 0.021 |
| DBP, mmHg | 66.00 (55.00, 79.00) | 67.00 (56.00, 80.00) | 66.00 (56.00, 79.00) | 64.00 (53.00, 78.00) | <0.001 |
| SBP, mmHg | 121.00 (104.00, 139.00) | 125.00 (108.00, 144.00) | 121.00 (105.00, 138.50) | 116.00 (100.00, 135.00) | <0.001 |
| **Laboratory** |  |  |  |  |  |
| Glucose, mg/dL | 164.00 (124.00, 228.00) | 152.00 (118.00, 199.00) | 163.00 (125.00, 227.00) | 182.00 (130.00, 257.25) | <0.001 |
| RBC, ×10^12^/L | 3.48 (2.94, 4.06) | 3.53 (3.00, 4.13) | 3.47 (2.97, 4.05) | 3.41 (2.88, 4.02) | <0.001 |
| WBC, ×10^9^/L | 11.80 (8.30, 16.60) | 10.30 (7.70, 13.90) | 11.80 (8.40, 16.40) | 13.60 (9.10, 19.40) | <0.001 |
| Hemoglobin | 10.20 (8.60, 11.90) | 10.30 (8.70, 12.00) | 10.20 (8.70, 11.80) | 10.00 (8.50, 11.70) | 0.022 |
| Anion gap, mmol/L | 15.00 (12.00, 18.00) | 14.00 (12.00, 17.00) | 15.00 (12.00, 17.00) | 16.00 (13.00, 20.00) | <0.001 |
| Calcium total, mmol/L | 8.30 (7.80, 8.80) | 8.50 (8.00, 8.90) | 8.30 (7.80, 8.80) | 8.20 (7.60, 8.70) | <0.001 |
| Chloride, mmol/L | 103.00 (99.00, 107.00) | 103.00 (99.00, 107.00) | 103.00 (98.00, 107.00) | 104.00 (99.00, 108.00) | 0.012 |
| Potassium, mmol/L | 4.30 (3.80, 4.80) | 4.20 (3.80, 4.70) | 4.30 (3.90, 4.80) | 4.40 (3.80, 4.90) | <0.001 |
| Sodium, mmol/L | 138.00 (135.00, 141.00) | 138.00 (135.00, 141.00) | 138.00 (135.00, 141.00) | 138.00 (134.00, 141.00) | 0.013 |
| ALT, IU/L | 29.00 (16.00, 75.00) | 25.00 (15.00, 53.00) | 29.00 (16.00, 66.50) | 38.00 (19.00, 132.78) | <0.001 |
| AST, IU/L | 42.00 (24.00, 107.50) | 32.00 (20.00, 63.00) | 41.00 (24.00, 89.00) | 59.50 (30.00, 213.25) | <0.001 |
| Creatinine, mg/dL | 1.30 (0.90, 2.20) | 1.25 (0.90, 2.20) | 1.30 (0.90, 2.10) | 1.40 (0.90, 2.20) | <0.001 |
| BUN, mg/dL | 27.00 (17.00, 46.00) | 26.00 (16.00, 46.00) | 27.00 (18.00, 47.00) | 29.00 (18.00, 48.00) | 0.005 |
| Albumin, g/dL | 3.00 (2.60, 3.40) | 3.30 (2.90, 3.60) | 3.00 (2.60, 3.40) | 2.80 (2.40, 3.20) | <0.001 |
| Lactate, mmol/L | 1.80 (1.20, 2.80) | 1.10 (0.90, 1.30) | 1.80 (1.50, 2.10) | 3.50 (2.80, 5.00) | <0.001 |
| LAR | 0.61 (0.41, 0.97) | 0.34 (0.29, 0.41) | 0.61 (0.54, 0.69) | 1.25 (0.97, 1.79) | <0.001 |
| Charlson | 7.00 (5.00, 9.00) | 7.00 (5.00, 9.00) | 7.00 (5.00, 9.00) | 7.00 (5.00, 9.00) | 0.006 |
| SIRS score | 3.00 (2.00, 3.00) | 2.00 (2.00, 3.00) | 3.00 (2.00, 3.00) | 3.00 (2.00, 4.00) | <0.001 |
| OASIS | 34.00 (28.00, 40.00) | 31.00 (26.00, 38.00) | 33.00 (28.00, 39.00) | 36.00 (31.00, 42.00) | <0.001 |
| GCS score | 15.00 (13.00, 15.00) | 15.00 (14.00, 15.00) | 15.00 (13.00, 15.00) | 15.00 (13.00, 15.00) | <0.001 |
| SOFA score | 6.00 (4.00, 9.00) | 5.00 (3.00, 7.00) | 5.00 (3.00, 8.00) | 8.00 (5.00, 11.00) | <0.001 |
| **Comorbidities, n (%)** |  |  |  |  |  |
| Hypertension | 2,196 (40.2%) | 718 (39.3%) | 740 (40.8%) | 738 (40.5%) | 0.611 |
| Chronic kidney disease | 1,808 (33.1%) | 647 (35.4%) | 602 (33.2%) | 559 (30.7%) | 0.011 |
| Hyperlipidemia | 2,702 (49.5%) | 923 (50.5%) | 931 (51.3%) | 848 (46.6%) | 0.010 |
| Coronary heart disease | 2,589 (47.4%) | 883 (48.3%) | 885 (48.8%) | 821 (45.1%) | 0.056 |
| Myocardial infarct | 769 (14.1%) | 249 (13.6%) | 262 (14.4%) | 258 (14.2%) | 0.771 |
| Heart failure | 2,298 (42.1%) | 833 (45.6%) | 763 (42.0%) | 702 (38.6%) | <0.001 |
| Cerebral vascular accident | 552 (10.1%) | 182 (10.0%) | 179 (9.9%) | 191 (10.5%) | 0.792 |
| **Interventions, n (%)** |  |  |  |  |  |
| Mechanical ventilation | 2,717 (49.7%) | 841 (46.0%) | 883 (48.7%) | 993 (54.6%) | <0.001 |
| CRRT | 585 (10.7%) | 152 (8.3%) | 167 (9.2%) | 266 (14.6%) | <0.001 |
| **Medications, n (%)** |  |  |  |  |  |
| Insulin | 3,100 (56.7%) | 1,013 (55.4%) | 1,055 (58.1%) | 1,032 (56.7%) | 0.256 |
| Glucocorticoid | 1,635 (29.9%) | 524 (28.7%) | 524 (28.9%) | 587 (32.3%) | 0.029 |
| Vasopressor | 3,471 (63.5%) | 987 (54.0%) | 1,140 (62.8%) | 1,344 (73.8%) | <0.001 |
| BMI, body mass index; DBP, diastolic blood pressure; SBP, systolic blood pressure; WBC, white blood cell; RBC, red blood cell; ALT, alanine aminotransferase; AST, aspartate aminotransferase; BUN, blood urea nitrogen; LAR, lactate to albumin ratio; SIRS, systemic inflammatory response syndrome; OASIS, oxford acute severity of illness score; GCS, Glasgow coma scale score; SOFA score, sequential organ failure assessment score; CRRT, continuous renal replacement therapy; ICU, intensive care unit. | | | | |  |
