# Supplementary material for: Association Between the Lactate‐to‐Albumin Ratio and ICU/In‐Hospital Mortality in Critically Ill Patients With Comorbid Type 2 Diabetes Mellitus : A Cohort Study Utilizing the MIMIC‐IV Database
Source: Emerg Med Int. 2026 Apr 13;2026:2751114. doi: 10.1155/emmi/2751114 (PMC13072064; doi:10.1155/emmi/2751114)
Supplement: Supplementary file 5 — Supporting Information 5 Supporting Table S5 Comparison of in‐hospital and ICU‐related mortality among different groups. [file EMMI-2026-2751114-s010.docx]

Comparison of in-hospital and ICU-related mortality among different groups

| **Outcome, n (%)** | **Total (N=5463)** | **Group 1 (N=1828)** | **Group 2 (N=1815)** | **Group 3 (N=1820)** | **P value** |
| --- | --- | --- | --- | --- | --- |
| In-hospital mortality | 985 (18.03) | 213 (11.65) | 297 (16.36) | 475 (26.10) | <0.001 |
| ICU mortality | 696 (12.74) | 144 (7.88) | 202 (11.13) | 350 (19.23) | <0.001 |
| 30-day ICU mortality | 1204 (22.04) | 267 (14.61) | 376 (20.72) | 561 (30.82) | <0.001 |
| 90-day ICU mortality | 1665 (30.48) | 416 (22.76) | 531 (29.26) | 718 (39.45) | <0.001 |
| 365-day ICU mortality | 2245 (41.09) | 615 (33.64) | 751 (41.38) | 879 (48.30) | <0.001 |
