# Supplementary material for: Association Between the Lactate‐to‐Albumin Ratio and ICU/In‐Hospital Mortality in Critically Ill Patients With Comorbid Type 2 Diabetes Mellitus : A Cohort Study Utilizing the MIMIC‐IV Database
Source: Emerg Med Int. 2026 Apr 13;2026:2751114. doi: 10.1155/emmi/2751114 (PMC13072064; doi:10.1155/emmi/2751114)
Supplement: Supplementary file 6 — Supporting Information 6 Supporting Table S6 In‐hospital mortality by LAR Cut‐off Value. [file EMMI-2026-2751114-s006.docx]

In-hospital Mortality by LAR Cut-off Value

| **LAR Group** | **Number of Patients (n)** | **Number of In-hospital Deaths (n)** | **In-hospital Mortality (%)** |
| --- | --- | --- | --- |
| LAR < 2.10 | 5122 | 848 | 16.56 |
| LAR ≥ 2.10 | 341 | 137 | 40.18 |
| Total | 5463 | 985 | 18.03 |

LAR, lactate-to-albumin ratio
