# Supplementary material for: Association Between the Lactate‐to‐Albumin Ratio and ICU/In‐Hospital Mortality in Critically Ill Patients With Comorbid Type 2 Diabetes Mellitus : A Cohort Study Utilizing the MIMIC‐IV Database
Source: Emerg Med Int. 2026 Apr 13;2026:2751114. doi: 10.1155/emmi/2751114 (PMC13072064; doi:10.1155/emmi/2751114)
Supplement: Supplementary file 7 — Supporting Information 7 Supporting Table S7 Harrell’s C‐index of the prognostic model across different follow‐up periods. [file EMMI-2026-2751114-s007.docx]

Harrell's C-index of the prognostic model across different follow-up periods

| **Follow-up Period** | **Harrell’s C-index** | **95% Confidence Interval** |
| --- | --- | --- |
| 30-day mortality | 0.724 | 0.710–0.738 |
| 90-day mortality | 0.697 | 0.685–0.709 |
| 365-day mortality | 0.675 | 0.663–0.687 |
