# Supplementary figures and images for: Association Between the Lactate‐to‐Albumin Ratio and ICU/In‐Hospital Mortality in Critically Ill Patients With Comorbid Type 2 Diabetes Mellitus : A Cohort Study Utilizing the MIMIC‐IV Database
Source: Emerg Med Int. 2026 Apr 13;2026:2751114. doi: 10.1155/emmi/2751114 (PMC13072064; doi:10.1155/emmi/2751114)

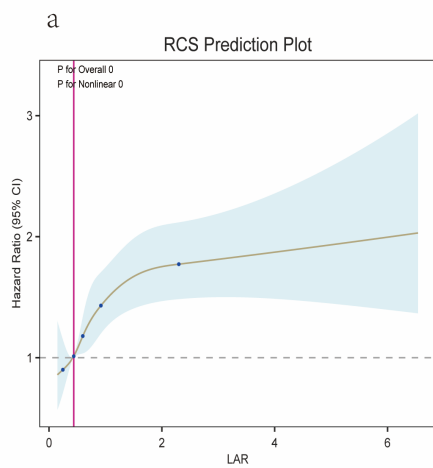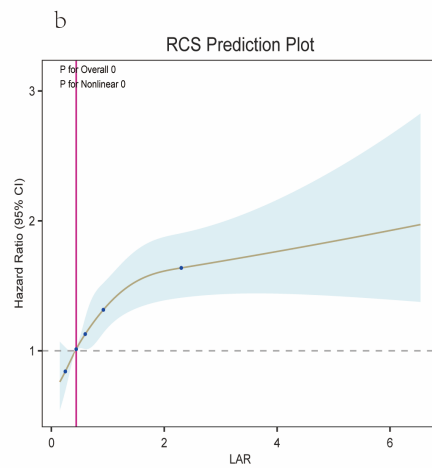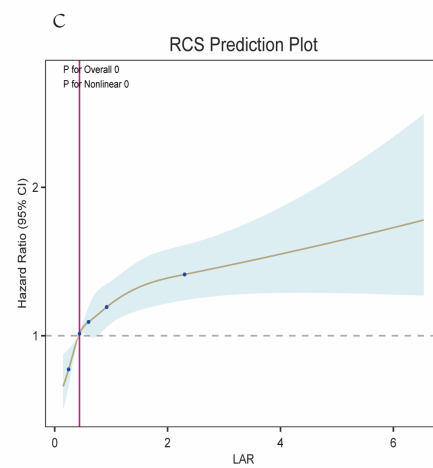

Supplement: Supplementary file 8 — Supporting Information 8 Supporting Figure 1 Restricted cubic spline function between LAR and 30‐day ICU mortality (a), 90‐day ICU mortality (b), and 365‐day ICU mortality (c), adjusted for age, CRRT, vasopressor, OASIS, SOFA score, mechanical ventilation, insulin, creatinine, AST, glucose, temperature, sodium, RBC, and WBC. LAR, lactate‐to‐albumin ratio. [file EMMI-2026-2751114-s008.pdf]

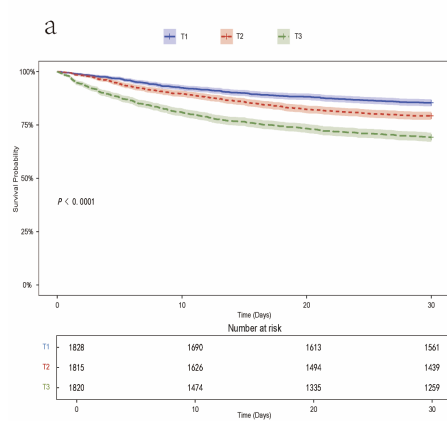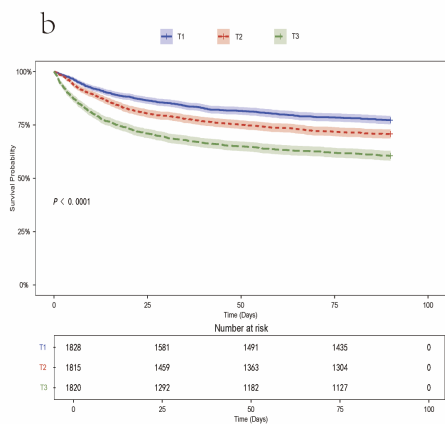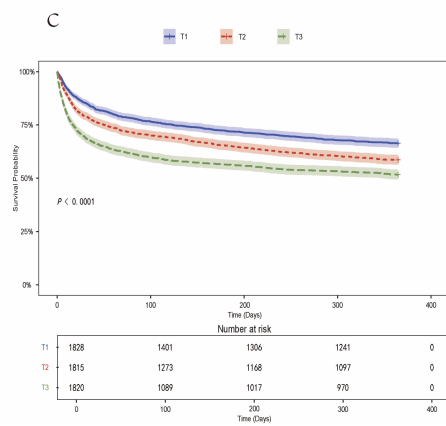

Supplement: Supplementary file 9 — Supporting Information 9 Supporting Figure 2 Kaplan–Meier survival curves for 30‐day (a), 90‐day (b), and 365‐day (c) all‐cause mortality according to tertiles of LAR. [file EMMI-2026-2751114-s009.pdf]

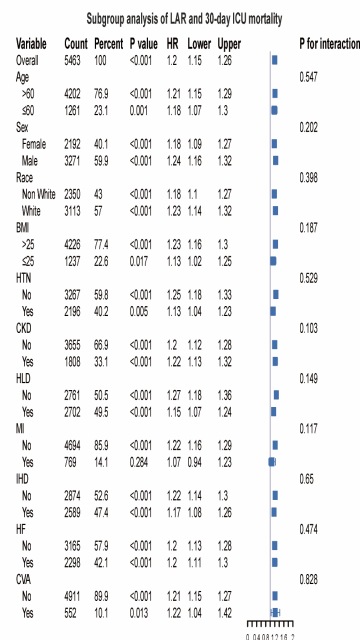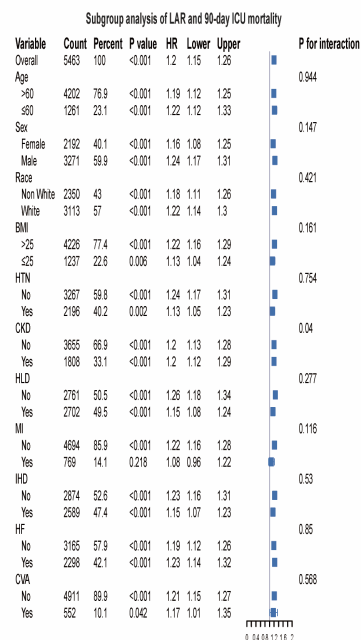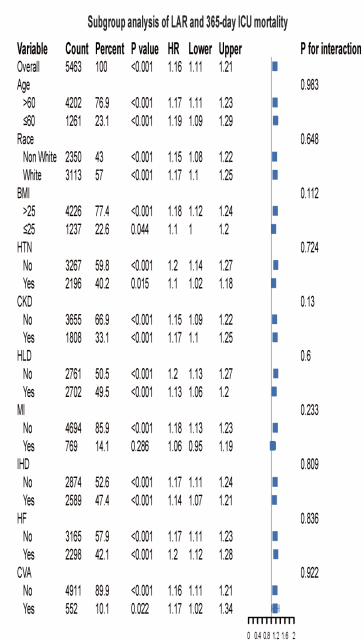

Supplement: Supplementary file 10 — Supporting Information 10 Supporting Figure 3 Subgroup analyses for the association of LAR with 30‐day ICU mortality (a), 90‐day ICU mortality (b), and 365‐day ICU mortality (c), adjusted for age, CRRT, vasopressor, OASIS, SOFA score, mechanical ventilation, insulin, creatinine, AST, glucose, temperature, sodium, RBC, and WBC. HR: Hazards ratio; LAR: lactate‐to‐albumin ratio; ICU: intensive care unit, BMI, body mass index, HTN, hypertension, CKD, chronic kidney disease, HLD, hyperlipidemia, MI, myocardial infarction, CHD, coronary heart disease, HF, heart failure, CVA, cerebrovascular accident. [file EMMI-2026-2751114-s005.pdf]
